# Proteome dataset of subcutaneous adipose tissue obtained from late pregnant dairy cows during summer heat stress and winter seasons

**DOI:** 10.1016/j.dib.2017.04.042

**Published:** 2017-05-04

**Authors:** M. Zachut, G. Kra, L. Livshitz, Y. Portnick, S. Yakoby, G. Friedlander, Y. Levin

**Affiliations:** a Department of Ruminant Science, Institute of Animal Sciences, Volcani Center, Rishon Lezion, Israel; b The Ilana and Pascal Mantoux Institute for Bioinformatics, The Nancy and Stephen Grand Israel National Center for Personalized Medicine, Weizmann Institute of Science, Rehovot, Israel; c De Botton Institute for Protein Profiling, The Nancy and Stephen Grand Israel National Center for Personalized Medicine, Weizmann Institute of Science, Rehovot, Israel

**Keywords:** Proteome, Adipose tissue, Heat stress, Dairy cow

## Abstract

Adipose tissue has a central role in the regulation of metabolism in dairy cows, and many proteins expressed in this tissue are involved in metabolic responses to stress (Peinado et al., 2012) [1]. Environmental heat stress is one of the main stressors limiting production in dairy cattle (Fuquay, 1981; West, 2003) [2], [3], and there is a complex interaction between heat stress and the transition period from late pregnancy to onset of lactation, which is manifested in heat-stressed late-gestation cows (Tao and Dahl, 2013) [4]. We recently defined the proteome of adipose tissue in peripartum dairy cows, identifying 586 proteins of which 18.9% were differentially abundant in insulin-resistant compared to insulin-sensitive adipose tissue (Zachut, 2015) [5]. That study showed that proteomic techniques constitute a valuable tool for identifying novel biomarkers in adipose tissue that are related to metabolic adaptation to stress in dairy cows. The objective of the present work was to examine the adipose tissue proteome under thermo-neutral or seasonal heat stress conditions in late pregnant dairy cows. We have collected subcutaneous adipose tissue biopsies from 10 late pregnant dairy cows during summer heat stress and from 8 late pregnant dairy cows during winter season, and identified and quantified 1495 proteins in the adipose tissues. This dataset of adipose tissue proteome from dairy cows adds novel information on the variety of proteins that are abundant in this tissue during late pregnancy under thermo-neutral as well as heat stress conditions. Differential abundance of 107 (7.1%) proteins was found between summer and winter adipose. These results are discussed in our recent research article (Zachut et al., 2017) [6].

**Specifications Table**

**Table:** 

Subject area	*Biology*
More specific subject area	*Adipose tissue proteomics*
Type of data	*Table*
How data was acquired	*Liquid Chromatography–Mass spectrometry: nanoAcquity+Q Exactive Plus*
Data format	*Raw, identifications, analyzed*
Experimental factors	*Adipose tissue biopsies were obtained from late pregnant dairy cows during the summer or winter seasons*
Experimental features	*Subcutaneous adipose tissue samples were collected from 10 late pregnant dairy cows during summer and 8 late pregnant dairy cows during winter, and were analyzed by Liquid Chromatography–Mass spectrometry following protein extraction. Differential expression was quantified using MS1 intensity based label-free.*
Data source location	*Rishon Lezion, Israel*
Data accessibility	*Data is with this article*

**Value of the data**

• This is the largest dataset of adipose tissue proteome from dairy cows.
• This dataset of adipose tissue proteome from dairy cows adds novel information on the variety of proteins that are abundant in this tissue during late pregnancy under thermo-neutral as well as heat stress conditions.
• Differential abundance of 107/1495 proteins in adipose of pregnant cows in summer.
• This study reveals several novel biomarkers of heat stress in cows׳ adipose tissue.

## 1 Experimental design, materials and methods

### 1.1 Animals and experimental procedures

The experimental protocol for the study was approved by the Volcani Center Animal Care Committee and was conducted at the Volcani Center experimental farm in Rishon Lezion, Israel. Thirty high-yielding, 261±5 days pregnant, non-lactating Israeli-Holstein dairy cows averaging 730±68 kg body weight (BW) and average lactation number 3.1 (range 2–6) participated in this study. Eighteen cows were in their dry period during August to September, which is the typical heat stress period in Israel (S group). The other 12 cows were in their dry period during December to January, which is the winter season in Israel (W group). All cows were group-housed in loose covered pens with adjacent outside yards and fed ad libitum once a day at 1100 h with standard diets both prepartum and postpartum according to NRC (1989) recommendations. The S cows were not cooled during late pregnancy, but they were exposed to five cooling sessions per day postpartum according to routine cooling management on the dairy farm [7].

### 1.2 Adipose tissue biopsies and sample processing

Adipose tissue biopsies were taken from each cow in late pregnancy (261±5 days of pregnancy, on average 14 days prepartum). Adipose tissue samples were taken from the subcutaneous fat pad around the pin bones. For protein extraction, ~40 mg adipose tissue was homogenized for 3 min with two metal beads (5 mm in diameter, Eldan Technologies, Petah Tikva, Israel) in 1 mL of pre-chilled lysis buffer containing 4% SDS, 0.1 M DTT, pH 7.6, and protease and phosphatase inhibitors (Sigma Aldrich, St. Louis, MO, USA). Samples were incubated for 1 h at 4 °C under continuous shaking, and then the homogenate was centrifuged at 20,000×*g* for 15 min at 4 °C to remove lipids and other particulate matter. The liquid phase was collected and stored at −80 °C.

### 1.3 Sample preparation for proteomic analysis

Protein concentration in each sample was determined using the bicinchoninic acid assay. Samples were then subjected to in-solution tryptic digestion using a modified filter-aided sample preparation (FASP) protocol. Samples were lysed for 3 min at 95 °C, and cell debris were removed by centrifugation (16,000*g*, 10 min). Lysed tissue (30 μL) was mixed with 200 μL urea buffer I (8 M urea in 0.1 M Tris–HCl pH 8.0), loaded onto 30-kDa molecular-weight-cutoff filters and spun down. A 200-μL aliquot of urea buffer II (2 M urea, pH 7.6–8.0; urea buffer I diluted 4X with 0.1 M Tris–HCl pH 7.6) was added to the filter unit and centrifuged (14,000×*g*, 40 min). Trypsin was added and the samples were incubated at 37 °C overnight. Digested proteins were centrifuged, acidified with TFA, and desalted in a solid-phase extraction column (Oasis HLB, Waters, Milford, MS, USA). Samples were stored at −80 °C until further analysis.

### 1.4 Liquid chromatography

Ultra LC–MS-grade solvents were used for all chromatographic steps. Each sample was subjected to split-less nano ultra-performance LC (UPLC) (10 K psi nanoAcquity, Waters). The mobile phases were: (A) H_2_O+0.1% (v/v) formic acid and (B) acetonitrile +0.1% formic acid. Samples were desalted online using a reverse-phase C18 trapping column (180-µm internal diameter, 20-mm length, 5-µm particle size; Waters). The peptides were then separated using an HSS T3 nano-column (75-µm internal diameter, 250-mm length, 1.8-µm particle size; Waters) at 0.35 µL/min. Peptides were eluted from the column into the MS using the following gradient: 4–35% solution B in 150 min, 35–90% B in 5 min, maintained at 95% for 5 min, and then back to initial conditions.

### 1.5 Mass spectrometry

The nano-UPLC was coupled online through a nano-ESI emitter (10-μm tip; New Objective, Woburn, MA) to a quadrupole orbitrap mass spectrometer (Q Exactive Plus, Thermo Scientific, USA) using a FlexIon nanospray apparatus (Thermo Scientific). Data were acquired in data-dependent acquisition mode using a Top20 method. The quadrupole isolation window was set to 1.6 mass units, MS1 resolution was set to 70,000 (at 400 m/z) with an AGC target of 3e6, and maximum injection time was set to 120 ms. MS2 resolution was set to 17,500 with an AGC target of 1e6 and a maximum injection time of 60 ms, and normalized collision energy was set to 26. Singly charged ions were excluded and dynamic exclusion was set to 60 s.

### 1.6 Data processing and analysis

Proteins were quantified by intensity-based label-free proteomics as described in Shalit et al. [8]. Raw data were processed as recently reported [8]; briefly, raw data were imported into Expressionist® software version 9.2.4 (Genedata). Data were filtered, smoothed, and aligned in retention time. This was followed by feature detection based on peak volume in retention time, *m/z* and intensity space, as well as isotopic clustering. A master peak list was generated from all MS/MS events and sent for database search using Mascot v2.5.1 (Matrix Sciences). Data were searched against the *Bos taurus* sequences in UniprotKB (http://www.uniprot.org/), version 2015_07, appended with 125 common laboratory-contaminating proteins for a total of 23,970 entries. Fixed modification was set to carbamidomethylation of cysteines and variable modification was set to oxidation of methionines. Search results were then imported into Scaffold version 3.5 (Proteome software) for filtering. False discovery rate was set to a maximum 1% at the protein level using the embedded ProteinProphet algorithm. Identifications were then imported to Expressionist. Protein grouping and quantification were conducted using an in-house script [8]. Protein quantification was based on the three most abundant peptides per protein, unless the protein was detected with two or one peptide.

Data were normalized based on the total ion current. Protein abundance was obtained by the iBAQ method (sum of all peptide intensities per protein divided by the theoretical number of tryptic peptides for that particular protein). Principal component analysis was used to assess the global integrity of the data and search for outlier samples.

### 1.7 Statistical analysis

Proteomics data, after logarithmic transformation, were analyzed by two-way ANOVA using Partek Genomic Suite (v6.6; St. Louis, MO). Our previous work showed that the degree of BW loss postpartum affects the proteome of adipose tissue in late-pregnant cows [5], and we therefore defined each cow as either high weight loss (HWL) or low weight loss (LWL) according to the percentage of BW loss from week 1 to week 5 postpartum as previously described [5], [9]. These subgroups were added to the statistical model. The two-way ANOVA examined the effects of season (S vs. W), subgroup (HWL vs. LWL), and their interaction. Differentially abundant proteins (DAPs) for each effect were determined by *P*-value <0.05 and an absolute fold change (FC) of at least ±1.5.

## 2 Data

This data describes the proteome of subcutaneous adipose tissues obtained from late pregnant dairy cows during summer heat stress or during the winter season. Adipose tissue has a central role in the regulation of metabolism in dairy cows, and many proteins expressed in this tissue are involved in metabolic responses to stress (Peinado et al., 2012) [1]. Environmental heat stress is one of the main stressors limiting production in dairy cattle (Fuquay, 1981; West, 2003) [2,3], and there is a complex interaction between heat stress and the transition period from late pregnancy to onset of lactation, which is manifested in heat-stressed late-gestation cows (Tao and Dahl, 2013) [4].Table 1 contains the list of 1495 identified and quantified proteins obtained by proteomic analysis.

This dataset of adipose tissue proteome from dairy cows adds novel information on the variety of proteins that are abundant in this tissue during late pregnancy under thermo-neutral as well as heat stress conditions. Differential abundance of 107 (7.1%) proteins was found between summer and winter adipose. These results are discussed in our recent research article (Zachut et al., 2017) [6].
